# London County Council and Tuberculosis Dispensaries

**Published:** 1920-12-04

**Authors:** 


### London County Council and Tuberculosis Dispensaries.

The London County Council have issued a series of instructions dealing with the working of the tuberculosis dispensary under their scheme for the treatment of tuberculosis in London. They suggest that consulting centres with physicians with special experience in tuberculosis and equipped with observation wards should be provided, if possible, at a number of London hospitals: that tuberculosis officers should have access to these beds, and that consultations upon the occupants of these beds and other patients brought by tuberculosis officers should take place at regular intervals, and that in the selection of consulting centres there should be a variation of existing arrangements with hospitals, each dispensary being, under modified arrangements, linked to a hospital which is also a recognised consulting centre. The fullest co-operation should be developed between the dispensary service and the school medical service. In each dispensary area in London the normal basis of staffing should provide for one tuberculosis officer for each 160 deaths a year in that area from tuberculosis; that part-time (preferably half-time) tuberculosis officers should assist in dispensary areas requiring, on the above-mentioned basis, the services of more than one whole-time tuberculosis officer, and that there should be one dispensary nurse (whole or part-time) for each tuberculosis officer (whole or part-time) in each dispensary area. The working week of each tuberculosis officer should normally be equivalent to twelve sessions of three hours each. At each dispensary at least one evening session in each week should ordinarily be held for the convenience of working men and women, and that, where practicable, a session be held each week on Saturday morning, or at some suitable time outside school hours, for children in attendance at school. Treatment as distinct from diagnosis consultation at the dispensary should as a rule be limited to patients whose continued treatment requires special knowledge or technical skill, and to those who are unable to obtain other adequate medical attendance. The practice of treating patients at the dispensaries on a large scale and over long periods with bottles of medicines, etc., and of giving medicine to ensure the attendance of patients, should be discouraged. In order to arrive at the earliest possible date at a definite diagnosis in doubtful cases there should be short periods of observation combined with more intensive study of each case, full use being made of the facilities and aids to diagnosis afforded by the consulting centre. In connection with each dispensary there should be adequate arrangements for following patients up for whose failure to continue in attendance at the dispensary no satisfactory reason has been ascertained. A tuberculosis officer should visit at least once the home of every notified case.

